# Effect of static magnetic fields and phloretin on antioxidant defense system of human fibroblasts

**DOI:** 10.1007/s11356-016-6653-x

**Published:** 2016-04-15

**Authors:** Katarzyna Pawłowska-Góral, Magdalena Kimsa-Dudek, Agnieszka Synowiec-Wojtarowicz, Joanna Orchel, Marek Glinka, Stanisław Gawron

**Affiliations:** Department of Food and Nutrition, School of Pharmacy with the Division of Laboratory Medicine in Sosnowiec, Medical University of Silesia, Jednosci 8, 41-200 Sosnowiec, Poland; Department of Molecular Biology, School of Pharmacy with the Division of Laboratory Medicine in Sosnowiec, Medical University of Silesia, Jednosci 8, 41-200 Sosnowiec, Poland; Institute of Electrical Drives and Machines KOMEL, 188 Rozdzienskiego Street, 40-203 Katowice, Poland

**Keywords:** Static magnetic field, QRT-PCR, Fibroblasts, Phloretin, Antioxidant defense system, Redox homeostasis

## Abstract

The available evidence from in vitro and in vivo studies is deemed not sufficient to draw conclusions about the potential health effects of static magnetic field (SMF) exposure. Therefore, the aim of the present study was to determine the influence of static magnetic fields and phloretin on the redox homeostasis of human dermal fibroblasts. Control fibroblasts and fibroblasts treated with phloretin were subjected to the influence of static magnetic fields. Three chambers with static magnetic fields of different intensities (0.4, 0.55, and 0.7 T) were used in the study. Quantification of superoxide dismutase 1 (*SOD1*), superoxide dismutase 2 (*SOD2*), glutathione peroxidase 1 (*GPX1*), microsomal glutathione S-transferase 1 (*MGST1*), glutathione reductase (*GSR*), and catalase (*CAT*) messenger RNAs (mRNAs) was performed by means of real-time reverse transcription PCR (QRT-PCR) technique. Superoxide dismutase (SOD), glutathione peroxidase (GPx), and catalase (CAT) activities were measured using a commercially available kit. No significant differences were found in *SOD1*, *SOD2*, *GPX1*, *MGST1*, *GSR*, and *CAT* mRNA levels among the studied groups in comparison to the control culture without phloretin and without the magnet. There were also no changes in SOD, GPx, and CAT activities. In conclusion, our study indicated that static magnetic fields generated by permanent magnets do not exert a negative influence on the oxidative status of human dermal fibroblasts. Based on these studies, it may also be concluded that phloretin does not increase its antioxidant properties under the influence of static magnetic fields. However, SMF-induced modifications at the cellular and molecular level require further clarification.

## Introduction

Living organisms exist in the Earth’s natural magnetic field (MF); therefore, they are genetically adapted to it. The flux density of Earth’s constant magnetic field varies between 30 and 60 μT. Added to that, artificial magnetic fields generated by permanent magnets are increasingly used in medical imaging, diagnostics, as well as in consumer devices, such as microphones, speakers, and home appliances (Saunders 2005; Ghodbane et al. 2013). MFs have been successfully used in medicine, mainly in treating disorders of the musculoskeletal, nervous, respiratory, cardiovascular and ocular systems, of the gastrointestinal tract, as well as in disorders of skin and soft tissue. The biological effects of MFs, such as the enhancement of soft tissue repair, or their anti-inflammatory and anti-edema properties have been established experimentally, and they constitute a scientific basis for clinical applications of magnetic fields (Markov 2007; Henry et al. 2008; Ekici et al. 2012). However, the application of MFs in daily life may carry the risk of functional disorders in cells, tissues, and biological systems.

Previous studies gave an ambiguous answer as to the potential harmfulness of the static magnetic field (SMF) to living organisms. It has been shown that SMF stimulation has little effect on cell growth and does not induce genotoxicity (Miyakoshi 2005). On the other hand, exposure to magnetic fields can increase the activity and lifetime of free radicals, which can lead to oxidative stress (Ghodbane et al. 2013). Enhanced reactive oxygen species (ROS) production can promote changes in the antioxidant enzyme activity, in the regulation of gene expression, and the intracellular calcium release. Oxidative stress also modifies the properties of the cell membrane and influences cell processes such as growth, proliferation, and death (Jouni et al. 2012). Moreover, increased oxidative stress may contribute to the pathogenesis of many diseases, including cancer and cardiovascular and skin diseases. Enzymatic and nonenzymatic antioxidant defense systems protect the body against the excessive production of reactive oxygen and nitrogen species (Aprioku 2013). Three levels of the antioxidant defense system have been described (Lobo et al. 2010; Jebakumar et al. 2012). The first line contains enzymes such as superoxide dismutase, catalase, glutathione peroxidase, and glutathione reductase. They contribute to prevent the formation of free radicals. The second line of defense involves lipid or water-soluble low-molecular-weight antioxidants, such as glutathione, ascorbic acid, tocopherol, and flavonoids, which scavenge the active radicals. The third line of defense includes repair enzymes, such as lipase, proteases, DNA repair enzymes, and transferases (Lobo et al. 2010; Jebakumar et al. 2012).

Apples and apple-derived products, widespread in the human diet, are an important source of different biological active substances such as phenolic compounds (Wang et al. 2014). Over the past few years, researches have demonstrated the unique and essential role of phloretin, a flavonoid naturally present in apples, in regulating many biological processes. Phloretin has been shown in in vitro studies to inhibit the growth of several cancer cells, including B16 murine melanoma, HL60 human leukemia cells, and HT-29 human colon cancer cells (Kobori et al. 1999; Schaefer et al. 2006). Apart from its anticancer properties associated with the induction of apoptosis through the activation of caspases and the promotion of BCL2-associated X (*Bax*) protein expression, the antioxidative, antimicrobial, and anti-inflammatory activities of phloretin have been documented (Yang et al. 2011; Chang et al. 2012; Barreca et al. 2014). The antioxidant activity of phloretin has been attributed to its dihydrochalcone structure. Furthermore, it was demonstrated that it inhibited the nuclear translocation of nuclear factor kappa-light-chain-enhancer of activated B cell (NF-κB) subunit p65 proteins and decreased phosphorylation in mitogen-activated protein kinase (MAPK) pathways (Chang et al. 2012). Also, phloretin has been shown to suppress matrix metalloproteinase-1 *(MMP-1*), the expression of which is involved in the breakdown of extracellular matrix, and it can protect against UV-induced skin damage (Leu et al. 2006; Shin et al. 2014). Therefore, phloretin is believed to have potential to serve as a preventive agent for ROS-related diseases.

Until now, there have only been a few reports giving an answer to the question on the effects of static magnetic fields generated by permanent magnets on human cells. The available evidence from in vitro and in vivo studies is deemed insufficient to draw conclusions about the potential health impact of static magnetic field exposure, because it demonstrates both positive and negative effects of SMF on cell functioning. Moreover, the mechanism of the influence SMFs exert on cells still remains unexplained. Therefore, the aim of the present study was to determine the influence of static magnetic fields of different flux densities (0.4, 0.55, and 0.7 T) and phloretin on the redox homeostasis of human dermal fibroblasts. The activities of antioxidant enzymes and the expression of genes encoding enzymes involved in the antioxidant defense system were evaluated.

## Material and methods

### Cell culture conditions

Normal human dermal fibroblasts (NHDF cell line) were obtained from the Clonetics (CC-2511; San Diego, CA, USA) and routinely maintained in the fibroblast basal medium (FBM; Lonza, Basel, Switzerland), supplemented with a human fibroblast growth factor-basic (hFGF-B), insulin, and gentamicin (FGM™ SingleQuots™; Lonza, Basel, Switzerland) at 37 °C in a 5 % CO_2_ incubator (Heraeus).

Both the cell number and viability were monitored by cell counting in the Countess TM Automated Cell Counter (Invitrogen, Carlsbad, CA, USA) after staining with 0.4 % trypan blue. The experiment was performed on cells in the logarithmic phase of growth under conditions of ≥98 % viability assessed by trypan blue exclusion. For the experiments, NHDF cells were used at four to six passages.

### Cytotoxicity

Method of 3-[4,5-dimethylthiazol-2-yl]-2,5-diphenyltetrazolium bromide (MTT) conversion was used to determine whether phloretin (Sigma-Aldrich, St Louis, MO, USA) at concentrations between 10^−8^ and 10^−3^ M was toxic to the fibroblast cell cultures. Phloretin was prepared as stock solution in dimethyl sulfoxide (DMSO; Sigma-Aldrich, St Louis, MO, USA) and then diluted in culture medium. For all experiments, the final concentration of DMSO in the medium was 0.1 % (*v*/*v*). Viability of cells was evaluated after 24 and 72 h of exposure to phloretin. The effect of this flavonoid on cell viability was evaluated in two independent experiments.

In the MTT assay, the ability of the cells to convert MTT (Sigma-Aldrich, St Louis, MO, USA) indicates mitochondrial activity and in consequence cell viability. Normal human dermal fibroblasts were seeded into 96-well culture plates (Nunc, Wiesbaden, Germany) at a density of 5000 cells/well and were treated with phloretin for 24 and 72 h. For control samples, the same volume of DMSO without phloretin was added. MTT (0.25 mg/ml) was added to the medium for 3 h (37 °C) before the end of the experiment. After being washed with phosphate buffered saline (PBS), cells were lysed in 100 μl of dimethyl sulfoxide (Sigma-Aldrich, St Louis, MO, USA) which enabled the release of the blue reaction product—formazan. Absorbance at the wavelength of 540 nm was read on a microplate reader Wallac 1420 VICTOR (PerkinElmer, Waltham, MA, USA).

### Exposure of NHDF cells to static magnetic fields

Control fibroblasts and fibroblasts treated with phloretin were subjected to influence of static magnetic fields. Phloretin was used in a concentration 10^−5^ M because in the higher concentration, this compound induced cytotoxic effect as it was evidenced before in cell viability assays.

To evaluate the effects of static magnetic fields control and treated with phloretin, NHDF cells were placed in magnetic test chambers (patent P—396639, Gawron et al. 2012). The magnetic chambers used to culture cells in a static magnetic field consisted of a ferromagnetic yoke, which constituted the bottom and cover of the chambers and permanent magnets. The chambers were enclosed by lateral, front, and back walls; the front wall was fitted with a window. The window dimensions corresponded to the lateral dimensions of a culture flask. Nonmagnetic distance plates determined the inner dimensions of the chambers, which were matched to the culture flask dimensions.

The design of these test chambers allowed for uniform distribution of magnetic flux density over the measurement space of the flask. In our study, three chambers were used, with three different magnet sizes (6-, 11-, and 20-mm thick). The flux densities in the chambers were 0.4, 0.55, and 0.7 T, respectively. The control culture chamber was not equipped with permanent magnets (steel were used instead) (flux density of 0.0 T). The cultures were maintained in test chambers at 37 °C in a 5 % CO_2_ incubator (Heraeus) for 3 days (72 h).

Next, the cells were washed with PBS and cell numbers were monitored by cell counting in the Countess TM Automated Cell Counter (Invitrogen, Carlsbad, CA, USA) after staining with 0.4 % trypan blue. Cells were pelleted and frozen at −70 °C for 24 h until RNA extraction.

### RNA extraction

Total RNA was extracted using a TRIzol reagent (Invitrogen, Carlsbad, CA), according to the manufacturer’s instructions. RNA extracts were treated with DNase I (RNeasy Mini Kit, Qiagen, Valencia, CA) according to the manufacturer’s instructions. The quality of extracts was checked electrophoretically using 0.9 % agarose gel stained with ethidium bromide (Sigma-Aldrich, St. Louis, MO). The results were analyzed and recorded using the 1D Bas-Sys gel documentation system (Biotech-Fisher, Perth, Australia). RNA concentration was determined using a GeneQuant II RNA/DNA spectrophotometer (Pharmacia Biotech, Cambridge, UK).

### Quantitative RT-PCR assay

Gene expression of *SOD1*, *SOD2*, *GPX1*, *MGST1*, *GSR*, *CAT*, and *β*-*actin* was evaluated using real-time reverse transcription PCR (QRT-PCR) and SYBR Green I chemistry (SYBR Green Quantitect RT-PCR Kit; QIAGEN, Valencia, CA). The analysis was performed using an Opticon™ DNA Engine Continuous Fluorescence Detector (MJ Research, Watertown, MA). All samples were tested in triplicate. *β*-*actin* was also included to monitor the QRT-PCR efficiency, as an endogenous positive control of amplification and integrity of extracts. Wells containing no template were run as negative controls. Oligonucleotide primers, specific for *SOD1*, *GPX1*, *GSR*, *CAT*, were designed on the basis of reference sequences (GenBank accession No. NM_000454; NM_000581; NM_000637, and NM_001752, respectively) using Primer Express TM Version 2.0 software (PE Applied Biosystems, Foster City, CA) (Table 1). Oligonucleotide primers specific for *SOD2*, *MGST1*, and *β*-*actin* were described previously by Gottipati and Cammarata (2008), Zenkel et al. (2005), and Strzalka et al. (2008) (Table 1).

**Table 1:** Characteristic of primers used for real-time QRT-PCR

Gene	Sequence of primers	Length of amplicon (bp)	Tm (°C)
*SOD1*	Forward: 5′-TTGGGCAATGTGACTGCTGACAAA-3′ Reverse: 5′-GGGCGATCCCAATTACACCACAA-3′	208	79.0
*SOD2*	Forward: 5′-CTGATTTGGACAAGCAGCAA-3′ Reverse: 5′-CTGGACAAACCTCAGCCCTA-3′	199	81.6
*GPX1*	Forward: 5′-AATGTGGCGTCCCTCTGAGGCA-3′ Reverse: 5′-GCTCGTTCATCTGGGTGTAGTCCCG-3′	55	85.0
*MGST1*	Forward: 5′-ATTGGCCTCCTGTATTCCTTG-3′ Reverse: 5′-TAATCCCTCTGCTCCCCTCC-3′	311	80.2
*GSR*	Forward: 5′-AGAAATCATCCGTGGCCATGCA-3′ Reverse: 5′-ACCAACAATGACGCTGCGGC-3′	214	82.0
*CAT*	Forward: 5′-CCTATCCTGACACTCACCGCCATCG-3′ Reverse: 5′-GGATGCTGTGCTCCAGGGCAGA-3′	201	82.0
*β*-*actin*	Forward: 5′-TCACCCACACTGTGCCCATCTACGA-3′ Reverse: 5′-CAGCGGAACCGCTCATTGCCAATGG-3′	295	85.0

*bp* base pairs, *Tm* melting temperature

The thermal profile for one-step RT-PCR was as follows: reverse transcription at 50 °C for 30 min, denaturation at 95 °C for 15 min, and 40 cycles consisting of the following temperatures and time intervals: 94 °C for 15 s, 60 °C for 30 s, and 72 °C for 30 s. Each run was completed using melting curve analysis to confirm the specificity of amplification and the absence of primer dimers. RT–PCR products were separated on 6 % polyacrylamide gels and visualized with silver salts.

### Quantification of expression of target genes

Relative messenger RNA (mRNA) expression of *SOD1*, *SOD2*, *GPX1*, *MGST1*, *GSR*, and *CAT* was determined using the 2^−(ΔΔCt)^ method (Livak and Schmittgen 2001), with *β*-*actin* as a reference gene, where ΔCt = Ct of our gene of interest—Ct of *β*-*actin*. The reference gene was validated to determine that the expression of this gene was unaffected by the experimental treatment. Gene expression levels of *SOD1*, *SOD2*, *GPX1*, *MGST1*, *GSR*, and *CAT* in fibroblast-stimulated phloretin and SMF were normalized to the expression level in untreated and unexposed cells.

### Biochemical analyses

For biochemical analyses, cells were washed twice with ice-cold PBS. Next, fibroblasts were mechanically homogenized for 5 min using an Ultra-Turrax homogenizer (IKA Labortechnik, Staufen, Germany), in a flask placed on ice. The homogenization time was experimentally established by assessing the effectiveness of the homogenization under a microscope. All studied biochemical parameters were recalculated to 10^6^ cells.

#### Superoxide dismutase activity assay

Superoxide dismutase (SOD) activity was estimated using a commercially available kit, RANSOD (Randox Laboratories, Poland), according to the manufacturer’s instructions. This method employs xanthine and xanthine oxidase to generate superoxide radicals, which react with 2-(4-iodophenyl)-3-(4-nitrophenol)-5-phenyltetrazolium chloride (INT) to form a red formazan dye. The superoxide dismutase activity is then measured by the degree of inhibition of this reaction. The absorbance at 505 nm was recorded for the calculation of SOD activity. One unit (U) of SOD causes a 50 % inhibition of the rate of reduction of INT under the conditions of this assay.

#### Glutathione peroxidase activity assay

Glutathione peroxidase (GPx) activity was measured using a commercially available kit, RANSEL (Randox Laboratories, Poland), according to the manufacturer’s instructions. In this method, glutathione peroxidase catalyzes the oxidation of glutathione by cumene hydroperoxide. In the presence of glutathione reductase and NADPH, the oxidized glutathione is immediately converted to its reduced form with the concomitant oxidation of NADPH to NADP+. The decrease in absorbance at 340 nm was measured.

#### Catalase activity assay

Catalase (CAT) activity was measured using the Catalase Assay Kit (Cayman Chemical, MI, USA). The method is based on the reaction of CAT with methanol in the presence of an optimal concentration of H_2_O_2_. The formaldehyde produced is measured spectrophotometrically with 4-amino-3-hydrazino-5-mercapto-1,2,4-triazole (Purpald) as the chromogen. Purpald specifically forms a bicyclic heterocycle with aldehydes, which upon oxidation changes from colorless to purple (data not shown).

#### Lactate dehydrogenase activity assay

Lactate dehydrogenase (LDH) activity was measured using an assay kit (Sigma-Aldrich, St Louis, MO, USA) according to the manufacturer’s instruction. The reduction of NAD+ to NADH, which was catalyzed by lactate dehydrogenase, was exploited in this assay. The absorbance at 450 nm was recorded for the calculation of LDH activity. The LDH activity was reported as the percentage of the control value (data not shown).

### Statistical analyses

Statistical analyses were performed using Statistica 9.0 software (StatSoft, Tulsa, OK), and the level of significance was set at *p* < 0.05. Values were expressed as means and standard deviation (SD) of three independent experiments. The one-way ANOVA test and Tukey’s post hoc test were applied to evaluate differences in the expression of examined genes and in the activity of SOD, GPx, and CAT among studied groups.

## Results

### Effect of phloretin on NHDF viability

**Fig. 1:**
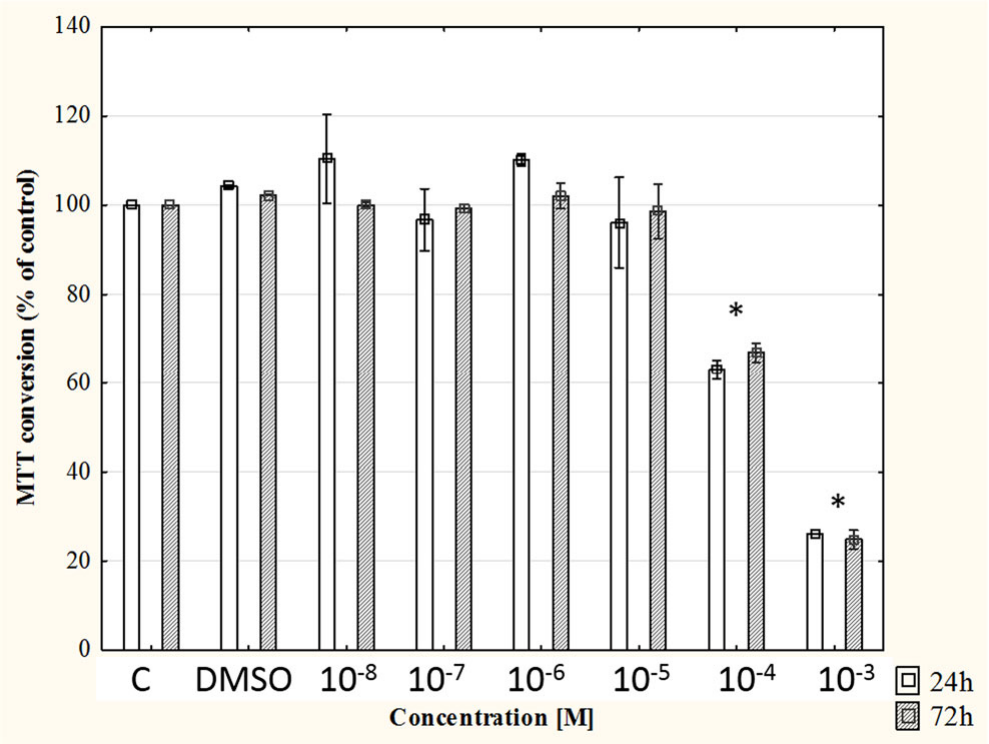
Cell viability in normal human dermal fibroblast cultures exposed to phloretin (between 10^−8^ and 10^−3^ M) for 24 and 72 h. *Each bar* represents the mean ± SD of two independent experiments. Statistical significance, *p < 0.05 vs. control (*C*)

According to the results of a cell viability test, phloretin was not cytotoxic to the normal human dermal fibroblasts in concentrations between 10^−8^ and 10^−5^ M (Fig. 1).

### Differences in *SOD1*, *SOD2*, *GPX1*, *MGST1*, *GSR*, and *CAT* expression level among the studied groups

There was no significant difference in the expression of *SOD1* among the control cells, cells exposed to SMF with 0.4, 0.55, and 0.7 T flux density, and cells treated with phloretin and exposed to SMFs (*p* > 0.05, one-way ANOVA test). No significant differences were also found for *SOD2*, *GPX1*, and *GSR* among the studied groups in comparison to the control culture without phloretin and without the magnet (flux density 0 T) (*p* > 0.05, one-way ANOVA test). However, the expression of *MGST1* was found to be significantly lower after the exposition of cells to SMFs with 0.4 and 0.55 T flux density than in the cells treated with phloretin and without the magnet (flux density 0 T) (*p* = 0.008 and *p* = 0.048, respectively, Tukey’s post hoc test). In turn, the mRNA level of *CAT* was significantly greater in fibroblasts exposed to SMF with 0.7 T flux density than in cells treated with phloretin and without the magnet (flux density 0 T) (*p* = 0.049, Tukey’s post hoc test) (Table 2).

**Table 2:** The relative expression of *SOD1*, *SOD2*, *GPX1*, *MGST1*, *GSR*, and *CAT* in NHDF cells exposed to static magnetic fields and in NHDF cells treated with phloretin, subjected to the influence of static magnetic fields of different intensities (0.4, 0.55, and 0.7 T) in comparison to the control culture without phloretin and without the magnet (flux density 0 T)

Relative expression						
	*SOD1*	*SOD2*	*GPX1*	*MGST1*	*GSR*	*CAT*
Control	1.01 ± 0.19	1.00 ± 0.02	1.01 ± 0.21	1.01 ± 0.14	1.00 ± 0.11	1.00 ± 0.12
Control + Ph	0.74 ± 0.17	1.13 ± 0.07	0.82 ± 0.24	1.34 ± 0.11	0.69 ± 0.09	0.57 ± 0.19
0.4 T	0.71 ± 0.28	0.94 ± 0.35	0.67 ± 0.20	0.84 ± 0.15*	0.73 ± 0.34	0.70 ± 0.18
0.55 T	0.81 ± 0.10	1.24 ± 0.29	0.82 ± 0.15	0.92 ± 0.33*	0.67 ± 0.12	0.80 ± 0.28
0.7 T	0.88 ± 0.16	1.14 ± 0.13	0.81 ± 0.20	0.94 ± 0.15	0.71 ± 0.12	0.95 ± 0.07*
0.4 T + Ph	0.86 ± 0.13	1.23 ± 0.22	0.82 ± 0.02	1.18 ± 0.05	0.57 ± 0.15	0.59 ± 0.12
0.55 T + Ph	0.95 ± 0.19	1.09 ± 0.23	0.85 ± 0.26	1.17 ± 0.17	0.79 ± 0.14	0.74 ± 0.08
0.7 T + Ph	0.89 ± 0.12	1.29 ± 0.17	0.84 ± 0.08	1.30 ± 0.20	0.62 ± 0.58	0.60 ± 0.12

*β*-*actin* was used as an endogenous control; means ± SD are presented

Statistical significance: *p < 0.05 vs. cells with phloretin and without magnet (flux density 0 T)

Control—control culture without phloretin and without magnet (flux density 0 T)

Control + Ph—control culture with phloretin (10^−5^ M) and without magnet (flux density 0 T)

0.4 T—culture without phloretin and with magnet thick 6 mm (flux density 0.4 T)

0.55 T—culture without phloretin and with magnet thick 11 mm (flux density 0.55 T)

0.7 T—culture without phloretin and with magnet thick 20 mm (flux density 0.7 T)

0.4 T + Ph—culture with phloretin (10^−5^ M) and with magnet thick 6 mm (flux density 0.4 T)

0.55 T + Ph—culture with phloretin (10^−5^ M) and with magnet thick 11 mm (flux density 0.55 T)

0.7 T + Ph—culture with phloretin (10^−5^ M) and with magnet thick 20 mm (flux density 0.7 T)

### The effect of the static magnetic fields and phloretin on the activities of antioxidant enzymes in NHDF cells

**Fig. 2:**
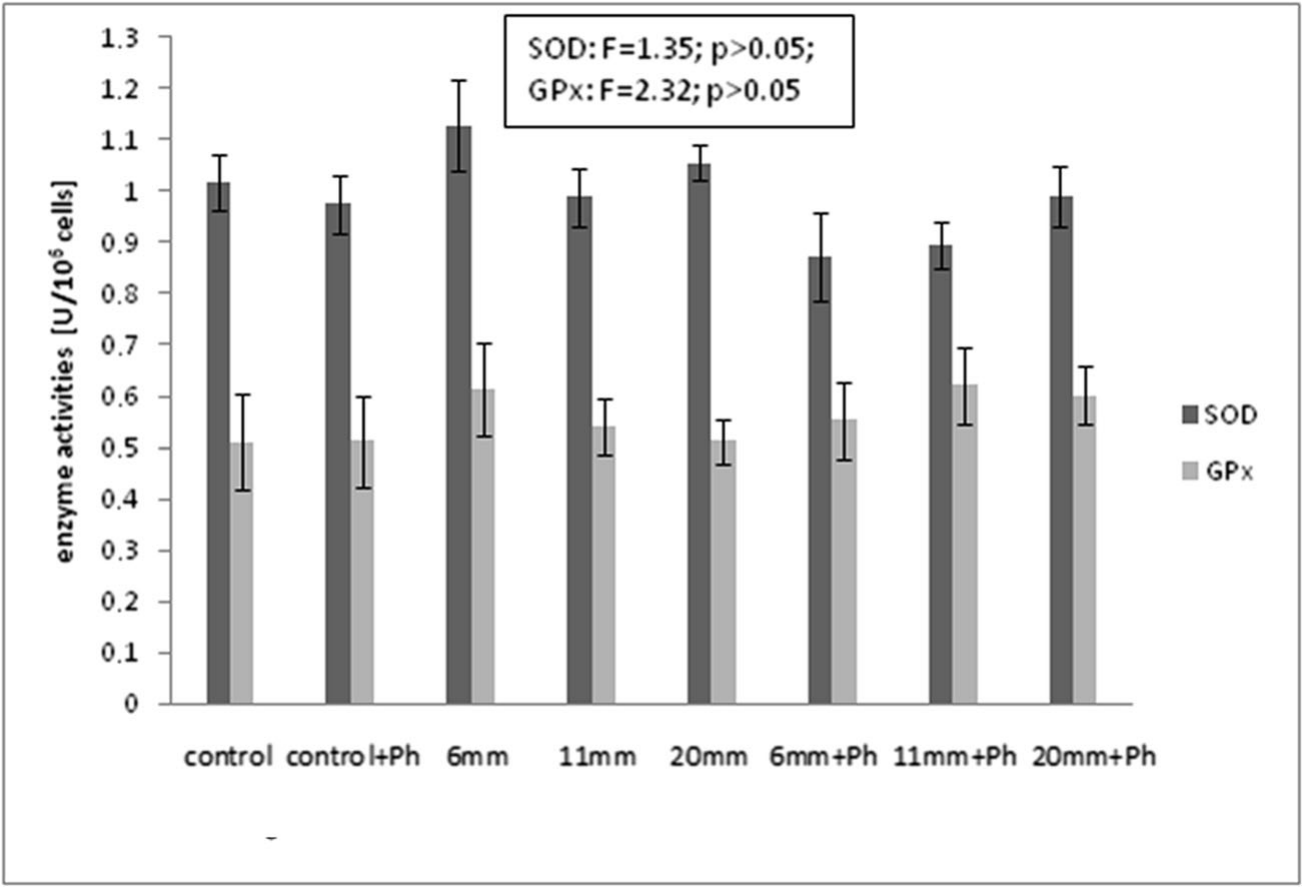
Effect of the static magnetic fields and phloretin on activities of antioxidant enzymes in NHDF cells. *Each value* represents the mean ± SD (*n* = 5); *p < 0.05 vs. control; #*p* < 0.05 vs. control + Ph; ^*p* < 0.05 vs. magnet control (6, 11, 20 mm); *control*—control culture without phloretin and without magnet (flux density 0 T); *control + Ph*—control culture with phloretin (10^−5^ M) and without magnet (flux density 0 T); *6 mm*—culture without phloretin and with magnet thick 6 mm (flux density 0.4 T); *11 mm*—culture without phloretin and with magnet thick 11 mm (flux density 0.55 T); *20 mm*—culture without phloretin and with magnet thick 20 mm (flux density 0.7 T); *6 mm + Ph*—culture with phloretin (10^−5^ M) and with magnet thick 6 mm (flux density 0.4 T); *11 mm + Ph*—culture with phloretin (10^−5^ M) and with magnet thick 11 mm (flux density 0.55 T); *20 mm + Ph*—culture with phloretin (10^−5^ M) and with magnet thick 20 mm (flux density 0.7 T)

In the cultures with phloretin and without static magnetic fields (control + Ph) and the cultures without phloretin and exposed to static magnetic fields (6, 11, 20 mm), there were no significant differences (*p* > 0.05) in SOD activities in comparison with the control cultures. In the cell cultures with phloretin and exposed to static magnetic fields with 0.4, 0.55, and 0.7 T flux density (6 mm + Ph; 11 mm + Ph; 20 mm + Ph), SOD activities were also not statistically significant in comparison with the control cultures and cultures without phloretin and without static magnetic fields (Fig. 2).

No significant differences (*p* > 0.05) were also found for GPx activities in the fibroblast cultures with phloretin and without SMF (control + Ph), in the cultures without phloretin and exposed to SMF (6, 11, 20 mm), and in fibroblasts treated with phloretin and exposed to static magnetic fields (6 mm + Ph, 11 mm + Ph, 20 mm + Ph) compared to the control (Fig. 2).

There were no changes in CAT activities in the studied fibroblast cultures in comparison with the control ones (data not presented).

## Discussion

The World Health Organization (WHO) recommended conducting in vitro studies to elucidate the nature of the interaction mechanisms and to help identify the effects of SMF. Such effects, if present, should then be further investigated in vivo for a proper risk evaluation of static magnetic field exposure (WHO 2006). In previous research, effects of static magnetic field have already been studied in vitro on various cell models. However, the results of these experiments are controversial and do not fully explain the possible consequences of static magnetic field exposure.

Static magnetic fields can influence the expression of specific genes on human and other mammalian cells, and the effects may depend on the duration of exposure and the magnetic flux density. The results of Laramee et al. (2014) revealed that the expression of heat shock protein (*HSP70*) in primary rat fibroblasts increased after the exposure to MFs of 1 to 440 mT. Heat shock proteins are a group of functionally related molecular chaperones that can be used as markers for cellular stress (Sõti et al. 2003). Genes related to cell stress defense mechanisms, including genes encoding molecular chaperones, antioxidant and pro-oxidant enzymes, and proteins involved in xenobiotic metabolism, may also be induced under free radical oxidative stress (Fulda et al. 2010). Based on the available data, Chekhun et al. (2012) concluded that the exposure of cells to SMF may cause disruption of free radical metabolism and the elevation of their concentration. In turn, free radical damage can be controlled by the suitable antioxidant defense systems. However, in our study, SMFs with different magnetic flux density levels (0.4, 0.55, and 0.7 T) had no or only minor effects on the expression of genes encoding enzymes involved in the antioxidant defense system: *SOD1*, *SOD2*, *GPX1*, *MGST1*, *GSR*, and *CAT* in comparison to the control culture. Additionally, no significant changes in the antioxidant enzyme activity in human fibroblasts were observed following the exposure to SMFs. Nevertheless, findings related to the influence of SMF on the cell antioxidant activity are contradictory. Lack of any SMF effect was demonstrated in murine fibroblasts (Glinka et al. 2013), but exposure to 6-mT static magnetic field induced oxidative stress in myelomonocytic leukemia cells (U937 cell line) (De Nicola et al. 2006). Furthermore, our previous studies conducted in vitro on murine fibroblasts also suggested that exposure to fluoride and a SMF improves the tolerance of cells to oxidative stress induced by fluoride ions (Kurzeja et al. 2013). However, this study was only performed at the protein level, not at the molecular level. Similarly, Traikov et al. (2009) indicated that exposure to 25-mT SMF decreased the levels of inflammatory and stress markers in rat blood plasma. Many researchers have also observed the lethal effects of moderate and strong SMFs combined with chemotherapy drugs on cancer cells such as K562 (human leukemia cells), HTB 63 (melanoma), HTB 77 IP3 (ovarian carcinoma), and CCL 86 (lymphoma, Raji cells) cell lines (Raylman et al. 1996; Sun et al. 2012). These conclusions point to the future possibility of clinical application for static magnetic fields in therapy.

The effects of exposure to static magnetic fields are often difficult to explain, largely due to differences in experimental parameters such as research conditions, exposure system, exposure times, and magnetic flux density of SMF. In this study, magnetic test chambers were used, in which cell culture flasks containing fibroblasts were placed. In the report of Ghodbane et al. (2011), however, compact electromagnets were applied. The results of Laramee et al. (2014) also demonstrated that the response to SMFs was dependent on experimental variables. These authors suggested that only longer exposure durations (12–48 h) demonstrate some significant response to SMF. However, in our study, SMF exposure was maintained for 72 h and we did not observe any significant changes in the redox homeostasis of human dermal fibroblasts. Moreover, in our study, the expression of genes encoding enzymes involved in the antioxidant defense system did not depend on flux density of static magnetic fields. The dependence on the experimental conditions was also observed by Lahbib et al. (2010), who found that, in rats, SMF effects on glucose and lipid metabolism were time-dependent. Likewise, it was observed that SMF inhibited IL-6 secretion in normal human colon myofibroblasts and this effect depended on the time of incubation (Gruchlik et al. 2012). However, it is very difficult to relate research results to human cells, because most research has been conducted on animal or cancer cells.

It was indicated that phloretin possibly plays a chemopreventive role through modulating the antioxidant and detoxification enzyme status (Anand and Suresh 2014). In the present study, we also strove to determine whether phloretin would increase its own antioxidant properties under static magnetic field influence. The addition of phloretin to the cell culture was manifested by a slightly higher transcriptional activity of *SOD2* and *MGST1* and a slightly lower expression of *SOD1*, *GPX1*, *GSR*, and *CAT*, in comparison with control cells. However, these differences were not significant. Likewise, static magnetic field exposure did not significantly change the expression of genes encoding enzymes involved in the antioxidant defense system in human dermal fibroblasts pretreated with phloretin. The present findings indicated that phloretin did not increase its antioxidant properties under exposure to SMFs with different flux density influences. Interestingly, the mRNA level of *MGST1* was significantly lower in cells treated with static magnetic fields of 0.4 and 0.55 T flux densities compared to the control cells treated with the tested flavonoid, whereas the mRNA level of *CAT* was significantly higher under static magnetic field exposure of 0.7 T flux density compared to control cells treated with phloretin. These observations may indicate that SMFs can diminish the influence of phloretin on the expression of the gene encoding a protein localized at the endoplasmic reticulum and outer mitochondrial membrane, where it is thought to protect these membranes from oxidative stress and the gene encoding a key antioxidant enzyme (Johansson et al. 2010).

In conclusion, our study indicated that SMFs generated by permanent magnets do not exert a negative influence on the oxidative status of human dermal fibroblasts. Based on these studies, it may be also concluded that phloretin does not increase its antioxidant properties under the influence of SMFs. However, SMF-induced modifications taking place at the cellular and molecular level require further clarification. Study of this issue will help to elaborate better treatment strategies for ROS-related diseases in the future.
